# Predictors of pulmonary embolism in hospitalized patients with COVID-19

**DOI:** 10.1186/s12959-023-00518-y

**Published:** 2023-07-03

**Authors:** Jeeyune Bahk, Abdul Rehman, Kam Sing Ho, Bharat Narasimhan, Hafiza Noor Ul Ain Baloch, Jiafang Zhang, Rowena Yip, Robert Lookstein, David J Steiger

**Affiliations:** 1 Department of Medicine, Mount Sinai Morningside and Mount Sinai West, Icahn School of Medicine at Mount Sinai, New York, NY USA; 2 Department of Medicine, Rutgers-New Jersey Medical School, Newark, NJ, ISA USA; 3 Division of Pulmonary and Critical Care, Department of Medicine, Mount Sinai West and Mount Sinai Beth Israel, Icahn School of Medicine at Mount Sinai, New York, NY 10019 USA; 4 Department of Biostatistics, Mount Sinai West and Mount Sinai Beth Israel, Icahn School of Medicine at Mount Sinai, New York, NY USA; 5 Department of Radiology, Mount Sinai Hospital, Icahn School of Medicine at Mount Sinai, New York, NY USA

**Keywords:** Pulmonary embolism, Venous Thromboembolism, Coagulopathy, D-dimer, Coronavirus disease 2019

## Abstract

**Background:**

High venous thromboembolism (VTE) rates have been described in critically ill patients with COVID-19. We hypothesized that specific clinical characteristics may help differentiate hypoxic COVID-19 patients with and without a diagnosed pulmonary embolism (PE).

**Methods:**

We performed a retrospective observational case-control study of 158 consecutive patients hospitalized in one of four Mount Sinai Hospitals with COVID-19 between March 1 and May 8, 2020, who received a Chest CT Pulmonary Angiogram (CTA) to diagnose a PE. We analyzed demographic, clinical, laboratory, radiological, treatment characteristics, and outcomes in COVID-19 patients with and without PE.

**Results:**

92 patients were negative (CTA-), and 66 patients were positive for PE (CTA+). CTA + had a longer time from symptom onset to admission (7 days vs. 4 days, p = 0.05), higher admission biomarkers, notably D-dimer (6.87 vs. 1.59, p < 0.0001), troponin (0.015 vs. 0.01, p = 0.01), and peak D-dimer (9.26 vs. 3.8, p = 0.0008). Predictors of PE included time from symptom onset to admission (OR = 1.11, 95% CI 1.03–1.20, p = 0.008), and PESI score at the time of CTA (OR = 1.02, 95% CI 1.01–1.04, p = 0.008). Predictors of mortality included age (HR 1.13, 95% CI 1.04–1.22, p = 0.006), chronic anticoagulation (13.81, 95% CI 1.24–154, p = 0.03), and admission ferritin (1.001, 95% CI 1-1.001, p = 0.01).

**Conclusions:**

In 158 hospitalized COVID-19 patients with respiratory failure evaluated for suspected PE, 40.8% patients had a positive CTA. We identified clinical predictors of PE and mortality from PE, which may help with early identification and reduction of PE-related mortality in patients with COVID-19.

## Introduction

Patients with severe coronavirus disease 2019 (COVID-19) develop coagulopathies associated with elevated D-dimer levels, venous thromboembolism (VTE), disseminated intravascular thrombosis (DIC), and bleeding [1]. Elevated D-dimer levels correlate with an increased risk of mortality [2]. High VTE rates in COVID-19 may be secondary to the prothrombotic and inflammatory state associated with COVID-19 through mechanisms such as cytokine storm, complement activation, and endotheliosis [3, 4]. There is autopsy evidence of both pulmonary macrothrombi and microthrombi, despite the use of prophylactic anticoagulation [5, 6]. VTE risk is the highest in patients admitted to intensive care units where prolonged immobilization, respiratory failure, and use of sedation and paralysis promote venous stasis leading to increased risks of thromboembolic events [7, 8]. VTE rates of 21–69% in critically ill patients with COVID-19 have been described, with rates greater than 20–30% occurring despite prophylactic anticoagulation [9]. Since the development of VTE is associated with increased mortality in patients with COVID-19, the diagnosis and treatment of thrombotic complications is of great importance [10–12].

An elevated D-dimer is used as a screening tool to rule out a pulmonary embolism (PE) in low or intermediate risk patients [13]. However, D-dimer lacks specificity for the diagnosis of PE in patients with COVID-19 [14]. Computed Tomography Angiography (CTA) is recommended when patients with COVID-19 develop increasing hypoxemia, hypotension, tachycardia without radiological evidence of advancing pneumonia [15], or when non-contrast chest CT findings do not provide sufficient explanation for the degree of hypoxia [16]. The aims of our study included defining predictors of PE in hospitalized hypoxic COVID-19 patients evaluated for a suspected PE. We hypothesized that specific demographic, clinical, and biochemical abnormalities may help differentiate hypoxic COVID-19 patients with and without a PE and define a cohort at an increased risk of a PE. In addition, we wanted to determine the predictors of mortality in patients who were diagnosed with a PE. Identifying predictors of PE and mortality in hospitalized patients with COVID-19 may help instruct clinicians to initiate therapy pending a radiological confirmation of PE, leading an early identification and reduction of PE-related mortality.

## Methods

### Study design, setting, and population

Our retrospective, observational, case-control study included all consecutive adult (> 18 years) patients hospitalized to one of four Mount Sinai Hospitals in New York City – the Mount Sinai Hospital, Morningside, West, and Beth Israel, between March 1 and May 8, 2020, with respiratory failure due to COVID-19 who received a CTA. The diagnosis of COVID-19 was confirmed by reverse transcriptase-polymerase-chain-reaction (RT-PCR) of nasopharyngeal or oropharyngeal specimens. Patients were defined as having a PE if the CTA was positive (CTA+), and PE negative if the CTA was negative for PE (CTA-). Thromboprophylaxis regimen was defined as subcutaneous low-molecular heparin (enoxaparin) 40 mg daily. The primary outcome was in-hospital PE, and further outcomes included mortality and hospital length of stay. A total of one hundred fifty-eight patients were identified. The institutional review board of Mount Sinai Health System approved this study. As no direct patient contact or intervention from the study group was needed, informed consent was waived. Researchers exclusively utilized de-identified data.

### Data collection

Clinical data was accessed via the electronic medical record system, EPIC, and relevant de-identified data extracted following review of patient medical charts. Patient demographics, co-existing medical conditions, clinical data including medications, vital signs, laboratory data, and imaging studies were collected. Coexisting medical conditions and presenting symptoms were obtained from physician documentation. All laboratory and imaging tests were performed at the discretion of the treating physician.

### Statistical analysis

All analyses were performed with R software (version 4.1.3; R Foundation for Statistical Computing, Vienna, Austria). Continuous variables are presented as means and standard deviations for normally distributed data or as medians and interquartile ranges for nonparametric data. Categorical variables are summarized as frequencies and percentages. Differences in distributions of characteristics of those with and without PE were analyzed using Student t test or Mann-Whitney U test for continuous variables and Chi-square or Fisher’s exact test for categorical variables. P-values were calculated with the use of two-sided exact tests and p ≤ 0.05 was considered to indicate statistical significance.

Multivariable logistic regression analysis was used to build the model to predict PE and stepwise selection was used for variable selection. Cox proportional hazard model was implemented to predict mortality in COVID-19 patients with PE and stepwise selection was used for variable selection. Firth penalized maximum likelihood estimation method was used to address rare event in the data and perfect separation.

To analyze the diagnostic performance of D-dimer tests for PE, a summary receiver operating characteristic curve was estimated with a multiple-threshold model, a multilevel random-effects model that considers sensitivity and specificity as functions of the thresholds and accounts for heterogeneity across studies, and the correlation of sensitivity with specificity [17]. We used data from patients who underwent CTA, as the presence or absence of PE could not be certain without CTA. The optimal cut offs were estimated by maximizing the Youden index under varying weights for sensitivity.

## Results

### Patients

A total of 158 patients who had a CTA were included. 66 (41.8%) patients had a positive CTA and 92 (58.2%) had a negative CTA. The median age of CTA + was 59 years, of whom 61% were male. The median age of CTA- was 64.5 years, of whom 52% were male. CTA + comprised more of White/Caucasians (33% vs. 13%), and fewer Asians (0% vs. 10%) compared to CTA- (p = 0.002). CTA + had significantly fewer patients with chronic obstructive pulmonary disease (COPD)/asthma (9% vs. 24%, p = 0.03) compared to CTA-. Average LOS for CTA + was 14.6 compared to 13.7 days for CTA- (p = 0.158). There was no statistically significant difference in the presence of parenchymal lung disease between the two groups (p = 0.817 by Chi-Square test). When radiological severity was categorized into mild, moderate, and severe, there was no statistically significant difference in the severity of lung involvement between the two groups (p = 0.394 by Chi-Square test). The baseline characteristics of both groups are summarized in Table 1.

Out of CTA+, 44% (n = 29) received therapeutic low-molecular heparin, 24% (n = 16) received unfractionated heparin, and 30% (n = 20) received a direct oral anticoagulant (DOAC) therapy.

**Table 1:** Demographic Data of Patients with and without a PE

Characteristics	Positive chest CTA consistent with a PE	Negative chest CTA ruling out a PE	Total	P-value
*Demographics*				
Age (yr), median (IQR) Males Females BMI (kg/m^2^), median (IQR)	59 (49-71) 40 (61) 26 (39) 28.6 (24.6-35.0)	64.5 (52.8-71) 48 (52) 44 (48) 27.6 (23.4-33.2)	62.5 (49.3-71.0) 88 (56) 70 (44) 28 (23.7-33.9)	0.40 0.37 0.32
*Race*				
Black or African American Hispanic White Asian Others	18 (27) 15 (23) 22 (33) 0 (0) 11 (17)	24 (26) 22 (24) 12 (13) 9 (10) 25 (27)	42 (27) 37 (23) 34 (22) 9 (6) 36 (23)	**0.002**
*Comorbidities*				
Hypertension Diabetes Hyperlipidemia Active malignancy COPD/asthma Coronary artery disease Congestive heart failure Atrial fibrillation Chronic kidney disease Chronic liver disease Cerebrovascular accident Myocardial infarction Autoimmune disease Previous gastrointestinal bleed Prior DVT/PE Oral contraceptive pill use	25 (38) 17 (26) 19 (29) 6 (9) 6 (9) 8 (12) 2 (3) 3 (5) 4 (6) 1 (2) 5 (8) 1 (2) 7 (11) 0 (0) 7 (11) 0 (0)	50 (54) 24 (26) 31 (34) 16 (17) 22 (24) 17 (19) 9 (10) 8 (9) 10 (11) 4 (4) 9 (10) 6 (7) 8 (9) 4 (4) 12 (13) 1 (1)	75 (48) 41 (26) 50 (32) 22 (14) 28 (18) 25 (16) 11 (7) 11 (7) 14 (9) 5 (3) 14 (9) 7 (6) 15 (10) 4 (3) 19 (12) 1 (1)	0.05 1.00 0.63 0.21 **0.03** 0.39 0.18 0.36 0.40 0.40 0.84 0.24 0.90 0.14 0.83 1.00
Smoking				
No	46 (70)	62 (67)	108 (68)	0.89
Active/prior	20 (30)	30 (33)	50 (32)	
Family history of DVT/PE	2 (3)	0 (0)	2 (1)	0.17
History suggestive of hyper-coagulable state	2 (3)	7 (8)	9 (6)	0.31
Recent surgery / immobilization	8 (12)	4 (4)	12 (8)	0.12
Chronic home anticoagulation				
None Lovenox/DOAC/coumadin Antiplatelet DAPT	63 (96) 3 (5) 9 (14) 1 (2)	83 (90) 9 (10) 20 (22) 3 (3)	146 (92) 12 (8) 29 (18) 4 (3)	0.36 0.28 0.64
Prophylactic anticoagulation				0.046
Prophylactic LMWH Therapeutic LMWH UFH DOAC	34 (37) 4 (4) 19 (21) 8 (9)	14 (21) 6 (9) 12 (18) 4 (6)	48 (30) 10 (60) 30 (20) 12 (76)	
Length of stay (mean days)	14.6	13.7		0.158
Severity of parenchymal lung disease				
None Mild Moderate Severe	10 27 20 9	12 30 30 20		0.817 0.394

### Clinical data: laboratory values, vital signs

Differences in laboratory values and vital signs at admission, at peak, and at the time of CTA are shown in Table 2. Compared to CTA-, CTA + had a longer time from symptom onset to admission (7 days vs. 4 days, p = 0.05), higher admission D-dimer (6.87 vs. 1.59, p < 0.0001), peak D-dimer (9.26 vs. 3.8, p = 0.0008), admission white blood cells (WBC) (10.2 vs. 7.45, p = 0.005), admission platelets (272.5 vs. 199.5, p = 0.001), admission total bilirubin (0.8 vs. 0.5, p = 0.003), admission direct bilirubin (0.4 vs. 0.3, p = 0.021), and admission troponin (0.015 vs. 0.01, p = 0.01). The median PESI score at the time of CTA was significantly higher for CTA + than CTA- (125.5 vs. 117.5, p = 0.006). No statistically significant difference was seen in lactate, BNP (b-type natriuretic peptide), troponin, blood pressure, oxygen saturations, or heart rate at the time of CTA between the two groups.

**Table 2:** Laboratory values and vital signs at admission, at peak, and at the time of CTA

Variables	Positive chest CTA consistent with a PE (n = 66)	Negative chest CTA ruling out a PE (n = 92)	Total	P-value
	Median (IQR)	Median (IQR)	Median (IQR)	
Time from symptom onset to admission (days) Admission D-dimer (ug/mL) Admission LDH (U/L) Admission CRP Admission WBC Admission Platelets Admission total bilirubin Admission direct bilirubin Admission troponin Admission BNP Peak IL-6 Peak LDH (U/L) Peak CRP (mg/L) Peak D-dimer Troponin at time of CTA Lactate at time of CTA PESI score at time of CTA SBP at time of CTA DBP at time of CTA SpO2 at time of CTA Heart rate at time of CTA	7 (2–14) 6.87 (2.10–20) 476.5 (353.75-6) 105.65 (68.8-199.5) 10.2 (6.5-14.78) 272.5 (195.25–349) 0.8 (0.6–1.2) 0.4 (0.3–0.5) 0.015 (0.01–0.097) 27.08 (10.3-95.48) 74.6 (27.23–130.5) 555 (377.5-696.25) 226.5 (124.7-302.3) 9.26 (2.60–20) 0.02 (0.01–0.1) 1.7 (1.3–2.9) 125.5 (113.3-140.5) 126 (111–141) 75 (66–82) 96 (94–98) 104 (86–114)	4 (2–7) 1.59 (0.79–3.4) 415.5 (308-591.8) 105 (42.01–192.9) 7.45 (5.9–10.4) 199.5 (158.8-261.3) 0.5 (0.4–0.9) 0.3 (0.2–0.5) 0.01(0.00-0.03) 25.8 (10.0-63.8) 61.15 (28.03–218.6) 558 (401.5–813) 196.2 (103.1-272.6) 3.8 (1.47–8.92) 0.01 (0.01–0.04) 1.6(1.2-2.0) 117.5 (100.8-133.5) 126( 114-136.5) 76 (70–83) 95 (93-97.3) 99.5 (86–110)	5 (2–10) 2.32 (1.14–10.7) 440 (319.5-619.5) 105.45 (55.4-198.4) 8.1 (6.1–12.3) 216 (170-316.3) 0.7 (0.4-1) 0.3 (0.2–0.5) 0.013 (0.01–0.05) 25.9 (10.0-71.2) 72.75 (26.675-174) 558 (386-789.5) 209 (113.04–281.4) 4.7 (1.71–17.5) 0.02 (0.01–0.06) 1.7 (1.2–2.3) 122 (105–136) 126 (112.8–138) 75 (68–83) 95 (93–98) 101 (86–113)	**0.05** **< 0.0001** 0.29 0.22 **0.005** **0.001** **0.003** **0.021** **0.01** 0.34 0.67 1.00 0.17 **0.0008** 0.26 0.26 **0.006** 0.76 0.15 0.37 0.45

Note – Continuous variables are presented as means and standard deviations for normally distributed data or as medians and interquartile ranges for nonparametric data. Differences in distributions of characteristics of those with and those without pulmonary embolism (PE) were analyzed using Student t test or Mann-Whitney U test. Bold indicates statistical significance (p < 0.05). CTA = computed tomography pulmonary angiography; LDH = lactate dehydrogenase; CRP = c-reactive protein; WBC = white blood cell; BNP = B-type natriuretic peptide; IL-6 = interleukin-6; PESI = pulmonary embolism severity index; SBP = systolic blood pressure; DBP = diastolic blood pressure

### Predictors of PE

The PE predictor model for patients hospitalized with COVID-19 was developed by initially performing univariate logistic regression analysis on all sixty-nine variables individually, which identified variables that were significantly associated with PE. Nine variables were selected to be included in the final model and are shown in Table 3. Multivariable logistic regression analysis was used to build the model to predict PE, and stepwise selection was used for variable selection based on AIC. Out of these, two statistically significant variables were identified: time from symptom onset to admission (OR = 1.11, 95% CI 1.03–1.20, p = 0.008), and PESI score at the time of CTA (OR = 1.02, 95% CI 1.01–1.04 (p = 0.008). In contrast, hypertension (OR = 0.34, 95% CI 0.13–0.85, p = 0.02) and COPD/asthma (OR = 0.22, 95% CI 0.06–0.68, p = 0.01) significantly predicted the absence of a PE.

**Table 3:** Regression analysis of predictors of PE in patients hospitalized with COVID-19 with and without a PE

Variable	Odds ratio [95% CI]	P-value
Peak D-dimer COPD/bronchial asthma Time from symptom onset to admission (days) PESI score at time of CTA Hypertension Admission total bilirubin Admission platelets Admission troponin Admission AST	1.06 (0.998–1.119) 0.22 (0.06–0.68) 1.11 (1.03–1.20) 1.02 (1.01–1.04) 0.34 (0.13–0.85) 2.27 (0.97–5.85) 1.003 (0.999–1.008) 3.67 (0.91–62.72) 0.99 (0.98-1.00)	0.06 **0.01** **0.008** **0.008** **0.02** 0.07 0.1 0.24 0.17

Note – Multivariable logistic regression analysis was used to build the model to predict pulmonary embolism and stepwise selection was used for variable selection. Bold indicates statistical significance (p < 0.05). COPD = chronic obstructive pulmonary disease; PESI = pulmonary embolism severity index; CTA = computed tomography pulmonary angiography; AST = aspartate transaminase

### Predictors of mortality in patients with PE

81% of patients were discharged, and overall mortality was 18.9%, including 22.8% (n = 21) in CTA- and 13.6% in CTA+ (n = 9). CTA- was sicker than CTA+. This was demonstrated by 12/21(57.1%) of CTA- requiring ICU admission vs. 4/9 (44.4%) of CTA+, of whom 9/21 (42.9%) of CTA- were intubated vs. 3/9 (33.3%) of CTA+, and 10/21 (47.6%) of CTA- required pressors vs. 1/9 (11.1%) of CTA+. Finally, 3/21 (14.2%) of CTA- underwent renal replacement therapies vs. 0 of CTA+. In addition, 8/21 (38.1%) of CTA- had a diagnosis of active malignancy vs. 1/9 (11.1%) of CTA+.

Variables for predictors of mortality in COVID-19 patients with PE were identified performing univariate Cox proportional hazard regression on all sixty-nine variables individually to select the variables that were significantly associated with mortality. Age, chronic home anticoagulation (AC), time from symptom onset to CTA, admission ferritin, C-reactive protein (CRP), blood urea nitrogen (BUN) were selected to be included in the multivariable Cox proportional hazard model and stepwise selection was used for variable section based on AIC, as shown in Table 4. Three statistically significant variables were identified: age (HR 1.13, 95% CI 1.04–1.22, p = 0.006), chronic home AC (13.81, 95% CI 1.24–154, p = 0.03), and admission ferritin (1.001, 95% CI 1-1.001, p = 0.01).

**Table 4:** Predictors of mortality among COVID-19 patients (univariate regression)

Variable	Hazard ratio [95% CI]	P-value
Age Chronic home AC Time from symptom onset to CTA (days) Admission ferritin Admission CRP Admission BUN	1.13 (1.04, 1.22) 13.81 (1.24, 154) 0.94 (0.88, 1.01) 1.001 (1, 1.001) 1.01 (0.99, 1.01) 1.03 (0.97, 107)	**0.006** **0.03** 0.07 **0.01** 0.07 0.07

Note – Cox proportional hazard model was implemented to predict mortality in COVID-19 patients with PE and stepwise selection was used for variable selection. Bold indicates statistical significance (p < 0.05). AC = anticoagulation; CTA = computed tomography pulmonary angiography; CRP = c-reactive protein; BUN = blood urea nitrogen

### Diagnostic performance of D-dimer for PE in patients with COVID-19

With respect to the admission D-dimer and the traditional cut-off level of 0.5, the sensitivity, specificity, positive predictive value (PPV), and negative predictive value (NPV) were 98.46%, 11.11%, 44.44% and 90.91%, respectively. For a cut-off level of 1.0, they were 90.77%, 30.0%, 48.36% and 81.82%, respectively.With respect to the Peak D-dimer cut-off level of 0.5, the sensitivity, specificity, PPV, and NPV were 98.46%, 4.44%, 42.67%, 80%. For a D-dimer cut-off of 1.0, they were 96.92%, 15.56%, 45.32% and 87.5%, respectively. These are summarized in Tables 5 and 6.

**Table 5:** Diagnostic performance of different cut-offs for admission D-dimer values for detecting pulmonary embolism in patients with COVID-19 (n = 155)

D-dimer cut-off (ng/ml)	Sensitivity	Specificity	PPV	NPV
0.50	98.46%	11.11%	44.44%	90.91%
1.00	90.77%	30%	48.36%	81.82%
1.25	86.15%	38.89%	50.45%	79.55%
2.50	61.54%	65.56%	56.34%	70.24%
5.00	50.77%	84.44%	70.21%	70.37%
7.50	49.23%	86.67%	72.73%	70.27%
10.00	46.15%	88.89%	75%	69.57%
15.00	35.39%	92.22%	76.67%	66.40%
20.00	30.77%	93.33%	76.92%	65.11%

**Fig. 1:**
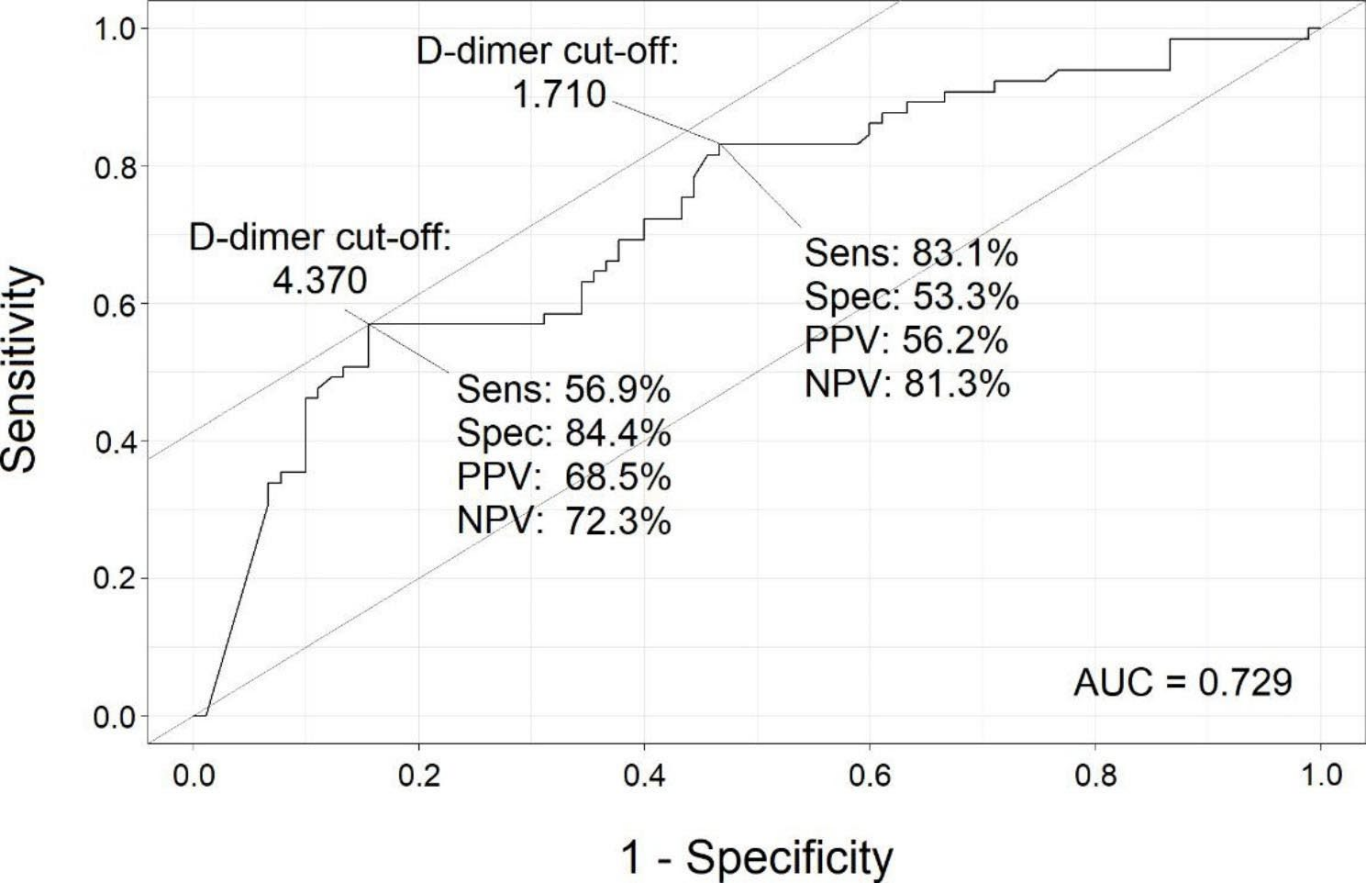
The Receiver Operating Characteristic Curves for Admission D-dimer Cut-off Levels

**Fig. 2:**
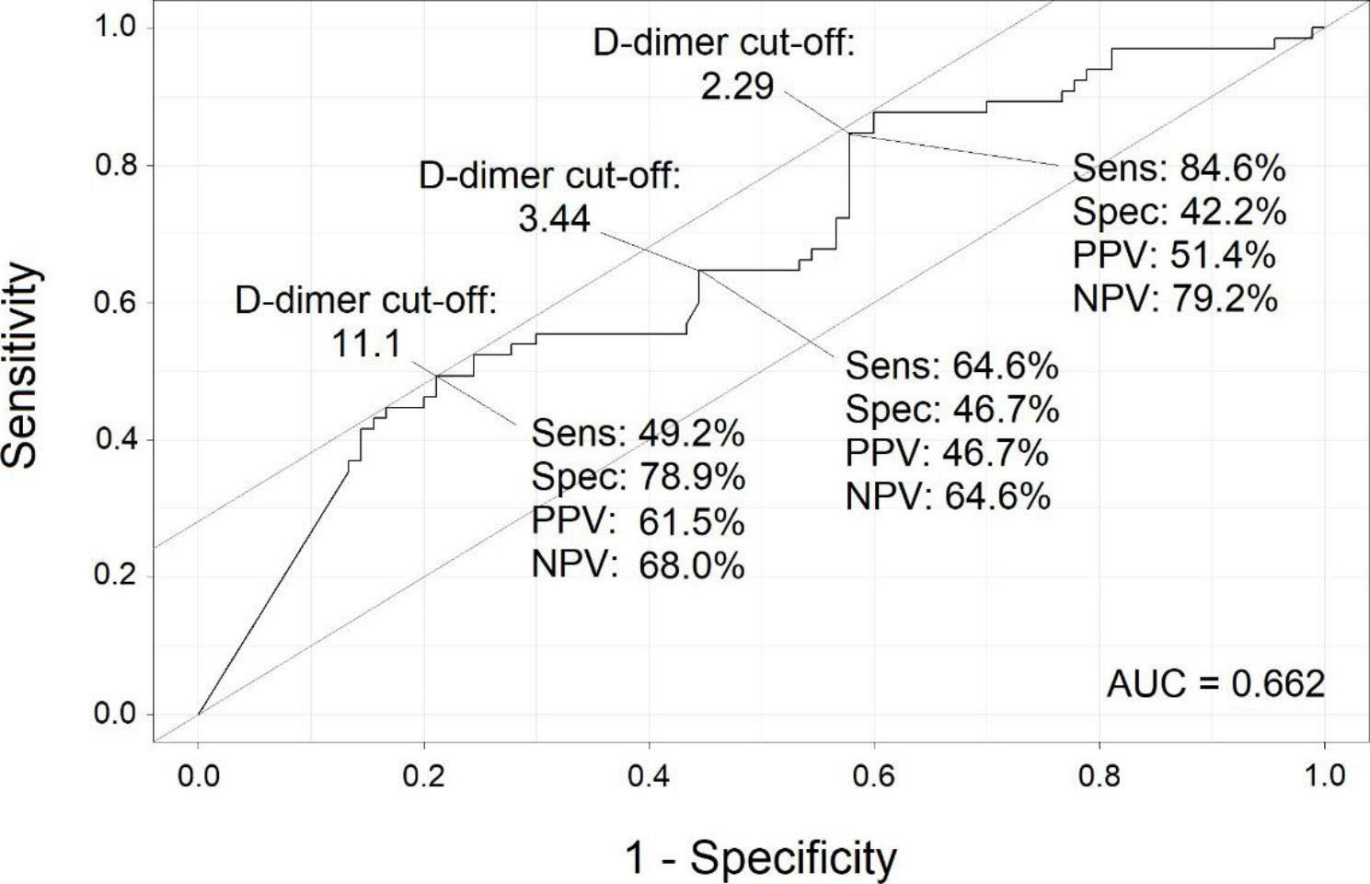
The Receiver Operating Characteristic Curves for Peak D-dimer Cut-off Levels

The summary receiver operating characteristic (ROC) curves yielded an area of 0.729 (Fig. 1) for admission D-dimer, and 0.662 for peak D-dimer (Fig. 2), suggesting cut-offs of D-dimer levels for PE diagnosis from 0 to maximum admission D-dimer in small incremental steps for PE diagnosis. For each single cut-off, sensitivity, specificity, PPV, NPV were calculated. Cut-off values that provided the best trade-off between sensitivity and specificity were selected.

For instance, using an admission D-dimer cut-off of 1.710, the sensitivity was 83.1% and specificity was 53.5%, whereas for an admission cut-off value of 4.370, the sensitivity of D-dimer tests for PE decreased (56.9%), but the specificity increased (84.4%). When admission D-dimer cut-off was raised, there was a reduction in sensitivity but an increase in specificity and PPV, as demonstrated in Table 5; Fig. 1.

Using a peak D-dimer cut-off of 3.44 (Fig. 2), the sensitivity was 64.6%, specificity was 46.7%, and PPV was 46.7%. Raised cut-off value to 11.1 was associated with decreased sensitivity (49.2%), but an increased specificity (78.9%) and PPV (61.5%), as demonstrated in Table 6; Fig. 2.

*PPV* = positive predictive value; *NPV* = Negative predictive value.

**Table 6:** Diagnostic performance of different cut-offs for peak D-dimer values for detecting pulmonary embolism in patients with COVID-19 (n = 155)

D-dimer cut-off (ng/ml)	Sensitivity	Specificity	PPV	NPV
0.50	98.46%	4.44%	42.67%	80%
1.00	96.92%	15.56%	45.32%	87.5%
1.25	93.85%	18.89%	45.52%	80.95%
2.50	76.92%	42.22%	49.02%	71.69%
5.00	56.92%	56.67%	48.68%	64.56%
7.50	55.39%	68.89%	56.25%	68.13%
10.00	49.23%	76.67%	60.38%	67.65%
15.00	44.62%	83.33%	65.91%	67.57%
20.00	35.39%	86.67%	65.71%	65%

*PPV* = positive predictive value; *NPV* = Negative predictive value

## Discussion

VTE is one of the most common complications in COVID-19 patients [18, 19]. In this retrospective study of patients hospitalized with acute COVID-19 who were evaluated for a possible acute PE, we describe demographics, clinical parameters, and laboratory abnormalities that were associated with patients diagnosed with a PE and were predictive of PE and mortality during hospitalization. Previous studies have described clinical and biochemical predictors of PE in patients with COVID-19 including D-dimer, inflammatory markers, and laboratory abnormalities including abnormal complete blood count, and changes in renal and hepatic function [20]. In common with some of these studies we determined that patients with PE, when compared to patients without PE, had a statistically significantly longer time from admission to PE symptom onset [20, 21], higher admission D-dimer and peak D-dimer [22–25], higher admission white blood cell count [26], and higher admission platelet count [26–28]. Of note, severe COVID-19 infection is associated with thrombocytopenia [27]. Additionally, we identified statistically significant higher admission total bilirubin, direct bilirubin [20], and admission troponin [25, 29, 30] in COVID-19 patients diagnosed with PE compared to those without PE. Moreover, median PESI score at the time of CTA was significantly higher for patients with a PE than for patients without a PE [26]. We did not see an increase in prevalence of more traditional risk factors for PE including concurrent cancer and a prior history of thromboembolism. This has been previously described [26, 31]. Increases in biomarkers including elevated WBC, platelet count [26, 27], elevated troponin [25, 29], and elevated prothrombotic biological markers including elevated D-dimer levels are associated with more severe COVID-19[32] and increased risk of PE [22, 25, 33, 34]. This emphasizes that inflammation contributes to the development of thrombosis in COVID-19 patients [35–[37]]. Although CRP levels have been used to predict progression and severity of COVID-19 [32], our study population CRP levels were not significantly different in COVID-19 patients with and without a PE, as has been previously described [20].

### Predictors of PE

Through univariate logistic regression analysis on sixty-nine variables individually, we were able to identify variables that were significantly associated with PE. We determined that predictors of PE included time from symptom onset to admission (OR = 1.11, 95% CI 1.03–1.20, p = 0.008), and PESI score at the time of CTA (OR = 1.02, 95% CI 1.01–1.04 (p = 0.008), whereas the presence of COPD/asthma (OR = 0.22, 95% CI 0.06–0.68, p = 0.01) and hypertension were inversely associated with predicting PE. Halpin described the prevalence of both asthma and COPD as being lower in patients with SARS-CoV-2 infection compared with the overall population prevalence of the diseases [38]. Moreover, it is also possible that a significant number of study patients in the COPD/asthma cohort had asthma, which is known to be protective against poor outcomes of COVID-19. Low rates of asthma have also been reported in a case series of patients hospitalized for COVID-19 [39], and asthma has been associated with lower mortality, specifically in patients with an eosinophilic asthma endotype [40].

A longer duration of hospitalization in COVID-19 patients with admission diagnosis of a PE compared to a shorter duration of stay in those who had a negative CTA has been previously described [20, 21]. The longer period of limited mobility of acute COVID-19 patients with more severe illness and increased length of stay, particularly in patients admitted to the ICU, may increase the risk of VTE [8]. Comorbidities have been previously described as risk factors for severe COVID-19 disease [41].

Increased PESI score at the time of CTA was associated with incidence of PE. PESI and simplified PESI score (sPESI) is a validated risk stratification tool to determine the 30-day and 90-day mortality of patients with an acute PE that uses eleven clinical and demographic criteria [42]. PESI and sPESI scores incorporate both vital signs and change in mental state, as well as history of cancer, heart failure (CHF), and chronic lung disease.

More severe COVID-19 in hospitalized patients is associated with increased inflammation with higher IL-6, D-dimer, Ferritin and LDH (20) with greater risk of VTE, admission to the ICU, and a higher mortality rate compared to hospitalized patients with less severe COVID-19 [6, 43]. Hospitalized COVID-19 patients who suffered barotrauma had higher inflammatory biomarkers including IL-6, LDH and D-dimer than admitted COVID-19 patients without barotrauma [44]. However, when we performed an analysis to determine whether more severe COVID-19, as manifest by a greater degree of infiltrates on Chest CT, was associated with a significant increased risk of PE, where radiological severity was categorized into mild, moderate, and severe, there was no statistically significant difference in the severity of lung involvement between the two groups (p = 0.394 by Chi-Square test).

### D-dimer

Traditionally, when evaluating a patient with a possible PE and the need for a diagnostic CTA, clinicians integrate pretest probability scores using Wells or Geneva criteria combined with D-dimer levels [30]. In patients with COVID-19, studies have not demonstrated any difference in Wells scores in patients suspected of PE with and without a diagnosed PE [45, 46]. Elevated D-dimer levels are common in patients admitted with COVID-19 [47] and increasing levels are associated with increased odds of mortality [48]. The elevated level is thought to be secondary to the proinflammatory milieu [49] induced by the COVID-19 viremia causing endothelial dysfunction, hyperviscosity, and hypoxia [50]. An elevation in D-dimer in severe COVID-19 may represent both the prothrombotic as well as the non-thrombotic inflammatory sequela of severe COVID-19. COVID-19 causes endothelitis, and activation of coagulation pathways resulting in a pro-coagulation state, and an influx of inflammatory cells. Endothelial injury reveals the thrombogenic basement membrane activating clotting. Proinflammatory cytokines including IL1-B, and TNF further activate endothelial cells promoting coagulation by expressing von Willebrand factor and fibrinogen, the binding of platelets, and increasing the expression of tissue factor via release of VEGF. High D-dimer levels, a fibrin degradation product, is an indirect marker of thrombotic activity and is part of the hosts response to coagulation that is promoted by COVID-19 related inflammation. In severe COVID-19 high levels of cytokines cause further endothelial cell dysfunction, inflammation, DIC and dilatation of the pulmonary capillary bed resulting in ARDS, and respiratory failure. Thus an elevated D-dimer in severe COVID-19 can represent significant inflammation promoting both coagulation and lung injury [50]. In a retrospective study of consecutive patients with acute PE that included COVID-19 positive and COVID-19 negative patients, although inflammatory (CRP) and prothrombotic markers ( APTT, Fibrinogen) were significantly elevated in the COVID-19 PE positive cohort, the D-dimer level was lower in the COVID-19 positive cohort compared to the negative cohort suggesting non-thrombotic mechanisms for D-dimer elevation [26]. A systematic analysis observed that COVID-19 patients with high D-dimer levels were at increased risks of severe disease, ARDS, and mortality [51]. Studies have been performed to identify an optimal threshold of D-dimer values to predict occurrence of PE and help guide the decision to further evaluate for the presence of PE. Study D-dimer levels were higher in patients with PE than those without PE as per previous studies [52, 53]. Using traditional cut-off D-dimer values of 0.5 and 1.0 for both admission D-dimer and peak D-dimer, both cut-offs were associated with a high sensitivity but unacceptably low specificity. The admission D-dimer threshold that was associated with a high sensitivity for diagnosing PE was 1.71 (sensitivity 83.1%, specificity 53.3%, PPV 56.2%, NPV 81.3%). The admission D-dimer cut-off level of 4.37 was associated with a lower sensitivity though with higher specificity and PPV. Peak admission D-dimer levels using a cut-off of 2.29, 3.44 and 11.1 were associated with increased specificity for diagnosing PE (42.2%, 46.7%, 78.9% respectively). Based on this, a peak D-dimer of 11.1 in a patient with possible PE should warrant an evaluation for PE.

### Predictors of Mortality

81% of patients were discharged, and overall mortality was 18.9%, including 22.8% in CTA- and 13.6% in CTA+. Age, elevated ferritin, and outpatient use of anticoagulation were predictors of mortality in patients with PE. Age as a predictor of worse outcome in COVID-19 is well-documented [54] and the incidence of PE increases with advancing age. The incidence of VTE is almost eight-times higher in individuals aged over 80 years than in the fifth decade of life [55]. PE-related mortality is the highest in 65–79 years age group [56]. The systemic inflammatory response secondary to COVID-19 results in an increase in inflammatory markers including ferritin leading to a hypercoaguable state [57]. Elevated ferritin has been reported to be associated with COVID-19 related thrombosis compared to thrombosis without COVID-19 [58]. Higher levels of ferritin are independent predictors of severe COVID-19 [59] and in-hospital mortality [60, 61]. Older patients with COVID-19 with elevated ferritin level demonstrate higher mortality than in patients with lower ferritin level [62]. An elevated ferritin level could be used as a biomarker to predict worse outcome in patients with COVID-19 and PE. Preadmission anticoagulation use was a predictor of mortality. Anticoagulation use was for a premorbid history of VTE and atrial fibrillation. Cardiovascular disease and atrial fibrillation are associated with an increased risk of mortality in COVID-19 [63]. It is proposed that SARS-CoV-2 infection alters cardiac cell-endothelial interaction, resulting in microvascular leakage, leading to release of inflammatory cytokines that effect atrial cellular electrophysiological stability [64]. Similarly, the higher mortality in CTA- compared to CTA+ (22.8% versus 13.6% respectively) is explained by the sicker cohort of CTA- who had a greater need for ICU admission, intubation, pressors, and renal replacement therapy (see Results).

### Anticoagulation

A greater number of PE positive patients (45.4%) did not receive any prophylaxis compared to PE negative patients (29%). Furthermore, 21% of PE positive patients received prophylactic LMWH versus 37% PE negative patients. However, a similar number of PE positive and PE negative patients received therapeutic anticoagulation (including therapeutic LMWH, full dose UFH, or a DOAC) before the diagnosis of PE (33.3% vs. 33.7%). The prophylactic regimens described in our patients reflect the lack of available hospital based standardized guidelines during the study period early during the COVID-19 pandemic. Present guidelines from the American Society of Hematology (ASH) suggest using prophylactic-intensity over intermediate-intensity anticoagulation for patients with COVID-19 related critical illness who do not have suspected or confirmed VTE [62]. Although VTE risk is higher in the critically ill COVID-19 patients, particularly in patients admitted to the ICU, standard prophylaxis is recommended, although there is evidence for the benefits of full dose anticoagulation in non-critically ill hospitalized COVID-19 patients [65, 66]. Our findings are of relevance for clinicians. There is a high prevalence of VTE in patients with COVID-19, and clinicians should consider patients with COVID-19 to be at an increased risk of VTE if they had prolonged hospital stay, and elevated biomarkers including an elevated D-dimer. Identifying these risk factors in patients hospitalized with COVID-19 may prompt the early initiation of empirical full dose anticoagulation pending results of a CTA.

### Limitations

Our study was an observational, retrospective study of a relatively small-sized cohort, exposing the study to possible selective and confounding bias. However, our data included consecutive patients evaluated for COVID-19 and a possible PE from four different institutions that serve a racially and socioeconomically diverse population in New York City. This study was performed during the first peak of the pandemic, where the prevalent strains of COVID-19 differ from present COVID-19 strains, the study population was unvaccinated, and the present standards of care for managing acute COVID-19 in hospitalized patients are different from the treatment protocols used during the study period. Further studies need to be performed to determine the applicability of our findings to patients infected with the newer strains of COVID-19.

## Conclusion

Patients hospitalized with COVID-19 are at increased risk of PE. We identified significant clinical predictors of PE and mortality from PE that can be used by physicians when empirically initiating full dose of anticoagulation for a possible PE pending radiological confirmation of a PE. Early initiation of anticoagulation in patients identified as at increased risk of developing PE may help reduce the risk of PE-related mortality in patients with COVID-19.

## Data Availability

The datasets used and/or analyzed during the current study are available from the corresponding author on reasonable request.
