# Correction: Predictors of pulmonary embolism in hospitalized patients with COVID-19

**DOI:** 10.1186/s12959-023-00531-1

**Published:** 2023-08-23

**Authors:** Jeeyune Bahk, Abdul Rehman, Kam Sing Ho, Bharat Narasimhan, Hafiza Noor Ul Ain Baloch, Jiafang Zhang, Rowena Yip, Robert Lookstein, David J Steiger

**Affiliations:** 1 Department of Medicine, Mount Sinai Morningside and Mount Sinai West, Icahn School of Medicine at Mount Sinai, New York, NY USA; 2 Department of Medicine, Rutgers-New Jersey Medical School, Newark, NJ, ISA USA; 3 Division of Pulmonary and Critical Care, Department of Medicine, Mount Sinai West and Mount Sinai Beth Israel, Icahn School of Medicine at Mount Sinai, New York, NY 10019 USA; 4 Department of Biostatistics, Mount Sinai West and Mount Sinai Beth Israel, Icahn School of Medicine at Mount Sinai, New York, NY USA; 5 Department of Radiology, Mount Sinai Hospital, Icahn School of Medicine at Mount Sinai, New York, NY USA

**Correction: Thrombosis J 21, 73 (2023)**

10.1186/s12959-023-00518-y

Following publication of the original article [1], it was noticed that the author name Hafiza Noor Ul Ain Baloch was incorrectly split into two different names due to a typesetting error.

The author group has been updated above and the original article has been corrected.

The publisher would like to apologize for any inconvenience this may have caused.
